# Type of Pegylated Interferon Matters: Another Milestone in the Treatment of Hepatitis C Virus Infection

**Published:** 2010-09-01

**Authors:** Ashwani Kumar Singal

**Affiliations:** 1 Division of Gastroenterology, Department of Internal Medicine University of Texas Medical Branch, Galveston, Texas, USA

Hepatitis C virus (HCV) infects 170 million people worldwide [1]. The infection becomes chronic in 85%-90% of cases with potential to cause cirrhosis, end-stage liver disease, and hepatocellular carcinoma (HCC) [2].

Treatment of HCV infection has evolved significantly over the last 20 years since the introduction of interferon-α in 1991. Initial studies using interferon were disappointing with response rates of less than 20%. Later, two important advances in the treatment of HCV infection, namely pegylation of interferon and introduction of ribavirin (RBV) in 2002, have revolutionized the treatment of HCV infection [3]. Pegylation is a process in which a polyethylene glycol (PEG) moiety is attached to the molecules used for the treatment [4]. This results in alteration of the pharmacokinetic, pharmacodynamic, and immunologic properties of the drug which in turn results in longer duration of action of the pegylated molecule allowing for using lower doses of the drug and better efficacy. Two pegylated molecules of interferon are pegylated interferon (PEG-IFN)-α2a (Pegasys) and -α2b (PegIntron). Both these molecules differ in terms of their physical characteristics and pharmacological properties (Table 1) [5]. One of the major differences between these two molecules is that PEG-IFN-α2b has a urethane bond which is unstable and is sensitive to hydrolysis. This results in release of interferon-α2b (the main therapeutic molecule) after injection of PEG-IFN-α2b. In contrast, PEG-IFN-α2a with an amide bond is chemically stable and the entire intact molecule has therapeutic effects [5]. PEG-IFN-α2a does not require any dose modification in the presence of renal insufficiency, in contrary to PEG-IFN-α2b which requires dose adjustment as the free interferon molecule released from PEG-IFN-α2b is mainly excreted by the kidneys [5]. PEG-IFN-α2b has a wide distribution throughout body fluids and tissues (Table 1) making its weekly dose adjustment based on patient's body weight (1.5 µg/kg). In contrast, PEG-IFN-α2a can be given in a fixed weekly dose of 180 µg [6].

**Table 1:** Physical and pharmacological characteristics of the two studied pegylated interferons.

**Characteristic**	**Peginterferon-α2a**	**Peginterferon-α2b**
**Structure**	12 kDa molecule	40 kDa branched molecule made of two 20 kDa molecules
**Positional isomers**	4	14
**Bond between pegylation and protein chain**	Stable amide bond	Unstable uretdane bond
**Storage**	Being stable, can be stored as solution for 2 years	Being unstable, stored as powder form and reconstituted immediately prior to injection
**Volume of distribution**	8–12 L/kg	1 L/kg
**50% absorption**	4–5 hrs	50 hrs
**Time to peak concentration**	20–40 hrs	80 hrs
**Metabolism**	70% liver; 30% kidneys	Mainly kidneys
**50% elimination**	40 hrs	65 hrs

So far, recommended treatment of HCV infection is a combination of PEG-IFN-α2a or -α2b in their respective doses plus RBV (given based on body weight for genotypes 1 and 4 while fixed dose of 800 mg for genotypes 2 and 3 infections) [7]. Treatment is determined to be successful with achievement of a sustained virologic response (SVR) which is defined as being HCV RNA negative after six months of completing the treatment. Achieving SVR is considered the best marker for permanent cure [7]. Over the last decade or so, since the discovery of the PEG-IFN, the data on the relative efficacy of PEG-IFN based on its type have been conflicting [8][9][10][11][12][13][14][15]. One of the largest study (IDEAL study) in which more than 3,000 patients infected with genotype 1 HCV infection were randomized into three treatment arms of either PEG-IFN-α2b 1.0 µg/kg/wk, PEG-IFN-α2b 1.5 µg/kg/wk or PEG-IFN-α2a 180 µg/kg/wk [11]. Although the end of treatment (EoT) response rate was higher with PEG-IFN-α2a as compared to PEG-IFN-α2b (64% vs. 53%), SVR was similar (41% vs. 40%). This was mainly due to higher relapse rate in those treated with PEG-IFN-α2a as compared to PEG-IFN-α2b (28% vs. 20%) [11]. Recently, two investigators in randomized controlled trials (RCTs) have shown better results with PEG-IFN-α2a as compared to PEG-IFN-α2b [14][15]. Rumi et al., randomized 447 patients into either PEG-IFN-α2a (n=223) or PEG-IFN-α2b (n=224); both groups also received RBV. Data on rapid virologic response (HCV RNA negative at wk 4), EoT response, and SVR were significantly higher with PEG-IFN-α2a than PEG-IFN-α2b (62% vs. 57%; 78% vs. 67%, and 66% vs. 54%, respectively) [14]. Another RCT by Ascione et al., also showed better efficacy with PEG-IFN-α2a (n=160) as compared to PEG-IFN-α2b (n=160). EoT response and SVR were in favor of PEG-IFN-α2a (84% vs. 64% and 69% vs. 54%, respectively). Data on rapid virological response (RVR) have not been reported in this study [15]. Both these studies reported similar types and frequencies of adverse events. Higher efficacy of PEG-IFN-α2a observed in these two studies, a finding in contrast to what found in the IDEAL study, can be explained by the fact that 40%-50% of patients included in these two studies had genotypes 2 or 3 HCV as compared to only genotype 1 HCV in the IDEAL study.

In this issue of Hepatitis Monthly, Alavian et al. report a systematic review of several RCTs comparing safety and efficacy of PEG-IFN-α2a and PEG-IFN-α2b [16]. Authors found that PEG-IFN-α2a is better and more effective than PEG-IFN-α2b in terms of achieving the EoT response and SVR. Although, the rate of neutropenia was higher with the PEG-IFN-α2a as compared to PEG-IFN-α-2b, proportion of patients requiring dose modification or drug withdrawal was similar with the two types of PEG-IFN administered [16]. Similar results have also been reported by another meta-analysis [17] (Table 2).

**Table 2:** Meta-analyses of the controlled randomized clinical trials comparing safety and efficacy of Peginterferon-α2a and Peginterferon-α2b plus ribavirin in the treatment of hepatitis C virus infection.

	**No. of RCTs^a^**	**No. of patients**	**RVR^a^**	**EoT response^a^**	**SVR^a^**	**Adverse events**	**Dose discontinuation**
**Alavian et al. [16]**	7	3,518	Not reported	1.67 (1.24–2.24)	1.38 (1.02–1.88)	1.50 (1.25–1.79)for neutropenia	0.78 (0.47–1.29)
**Awad et al[17]**	8	4,335	Not reported	Not reported	1.11 (1.04–1.19)	Not reported	0.79 (0.51–1.23)

^a^ RCT: randomized controlled trial; RVR: rapid virological response; EoT: end of treatment; SVR: sustained virologic response

What can be the reasons for this selective advantage with PEG-IFN-α2a? Stability of PEG-IFN-α2a with its resistance to hydrolysis makes its absorption as well as elimination slower than PEG-IFN-α2b [18]. These pharmacological differences may explain the availability of PEG-IFN-α2a for a longer period after injection as compared to PEG-IFN-α2b. Pharmacodynamic studies have shown that PEG-IFN-α2a is available at a maximum concentration for up to 168 hrs after injection as compared to only 72 hrs for PEG-IFN-α2b [8][18].

Demonstration of higher efficacy of PEG-IFN-α2a in comparison to PEG-IFN-α2b is another milestone in the evolution of treatment of HCV infection. Better SVR is likely to result in an improved outcome and long-term morbidity and mortality from HCV infection [19]. However, further studies comparing the two types of PEG-IFN with longer follow-up are needed to answer this question. Moreover, whether similar efficacy of PEG-IFN-α2a also applies to special populations such as children, non-responders to initial treatment, and those with HIV co-infection needs to be addressed in future studies [20].
